# Explicit motor sequence learning after stroke: a neuropsychological study

**DOI:** 10.1007/s00221-021-06141-5

**Published:** 2021-06-05

**Authors:** Cristina Russo, Laura Veronelli, Carlotta Casati, Alessia Monti, Laura Perucca, Francesco Ferraro, Massimo Corbo, Giuseppe Vallar, Nadia Bolognini

**Affiliations:** 1 Department of Psychology and Milan Center for Neuroscience-NeuroMi, University of Milano-Bicocca, Milan, Italy; 2 Department of Neurorehabilitation Sciences, Casa di Cura Policlinico, Milan, Italy; 3 Laboratory of Neuropsychology, IRCCS Istituto Auxologico Italiano, Milan, Italy; 4 Department of Neurorehabilitation Sciences, IRCCS Istituto Auxologico Italiano, Milan, Italy; 5 Department of Biomedical Sciences for Health, Università Degli Studi di Milano, Milan, Italy; 6 Riabilitazione Specialistica Neuromotoria - Dipartimento di Neuroscienze, ASST “Carlo Poma” di Mantova - Presidio di Riabilitazione Multifunzionale di Bozzolo, Mantua, Italy

**Keywords:** Motor learning, Affected hand, Finger tapping task, Stroke

## Abstract

Motor learning interacts with and shapes experience-dependent cerebral plasticity. In stroke patients with paresis of the upper limb, motor recovery was proposed to reflect a process of re-learning the lost/impaired skill, which interacts with rehabilitation. However, to what extent stroke patients with hemiparesis may retain the ability of learning with their affected limb remains an unsolved issue, that was addressed by this study. Nineteen patients, with a cerebrovascular lesion affecting the right or the left hemisphere, underwent an explicit motor learning task (finger tapping task, FTT), which was performed with the paretic hand. Eighteen age-matched healthy participants served as controls. Motor performance was assessed during the learning phase (i.e., online learning), as well as immediately at the end of practice, and after 90 min and 24 h (i.e., retention). Results show that overall, as compared to the control group, stroke patients, regardless of the side (left/right) of the hemispheric lesion, do not show a reliable practice-dependent improvement; consequently, no retention could be detected in the long-term (after 90 min and 24 h). The motor learning impairment was associated with subcortical damage, predominantly affecting the basal ganglia; conversely, it was not associated with age, time elapsed from stroke, severity of upper-limb motor and sensory deficits, and the general neurological condition. This evidence expands our understanding regarding the potential of post-stroke motor recovery through motor practice, suggesting a potential key role of basal ganglia, not only in implicit motor learning as previously pointed out, but also in explicit finger tapping motor tasks.

## Introduction

Motor recovery from stroke remains an open issue of interest for both neuroscientists interested in uncovering how our brain copes with a cerebral damage, and for clinical researchers interested in the development of effective therapy for motor rehabilitation. Hemiparesis, the most prominent motor sequelae after a unilateral brain damage, results from lesions to efferent motor pathways, and causes motor impairments in the side of the body contralateral to the side of the lesion (contralesional). About 40% of stroke survivors suffer from functional impairments and severe disability (Ward 2005), which often persists despite long-lasting standard rehabilitation care (i.e., physical, occupational and cognitive therapy). This has favored the spread of many rehabilitation treatments, mainly based on intensive motor practice (Krakauer 2015), and learning (Shishov et al. 2017), aimed at promoting the re-acquisition of lost motor skills.

In fact, the learning-based re-acquisition of compromised, or even lost, motor skills appears crucial for recovery from post-stroke hemiparesis (Wessel et al. 2015). As for the learning of new motor tasks in healthy adults, re-learning processes after brain damages are grounded on mechanisms of neuroplasticity (Warraich and Kleim 2010; Ward 2005). Critically, cortical plastic changes take place only when a learning component is involved, while simple motor practice, namely, a mere repetitive use of a limb, has almost null effects on neuroplasticity (Plautz et al. 2000). Motor re-learning after stroke improves motor functions by guiding performance amelioration over time, with an appreciable increase in accuracy, a decrease in response latencies, or both (Dobkin 2008). This amelioration, in turn, results from the better selection and execution of single movements, as well as from the grouping of motor memory items into larger units, or *chunks* (Boutin et al. 2013; Lungu et al. 2014).

Motor learning involves different stages: the acquisition of a simple motor skill starts with a “fast” improvement (“online” motor learning), which emerges during the initial practice session, and continues with an incremental amelioration, which requires a longer time to stabilize (“slow” learning) (Karni et al. 1998). An intermediate stage between these “fast” and “slow” learning phases is represented by processes taking place between sessions, with no further practice (“offline” motor learning) (Dayan and Cohen 2011; Doyon 2008). Both “online” and “offline” gains can be maintained over time, resulting in *retention* (Romano et al. 2010).

Decades of research on motor learning have favored the spread of many motor paradigms. With respect to post-stroke upper-limb hemiparesis, rehabilitation is mainly focused on the re-learning of serial movements. This is because serial (or sequential) behavior is crucial for activities of daily living and requires multiple movement elements to be integrated through practice into a single motor behavior (Doyon 2008).

Motor sequence learning tasks can be performed with either an “implicit” paradigm, in which participants are not informed of the presence of repeated patterns of movements, or an “explicit” paradigm (Fleming et al. 2017), in which participants are given a fixed sequence to learn and are aware of both the learning process and the sequential order of the elements (Dahms et al. 2020).

Many tasks have been used to investigate learning mechanisms, requiring different gross (e.g., moving a mouse) or fine (e.g., pressing a sequence of keys) movements. For instance, patients may be engaged in sequential visual isometric pinch tasks, which involve learning to control a force transducer, to move a cursor displayed on a computer screen (e.g., Reis et al. 2009; Saucedo Marquez et al. 2013). In serial reaction time tasks (SRTT), patients are involved in visuomotor implicit activities that require to respond by pressing keys to a series of stimuli presented at varying locations on a computer screen (for a review, see Kal et al. 2016). Worth mentioning, in stroke patients, spontaneous motor recovery is mainly associated with improvements of gross motor functions, while for recovering fine motor skills, specific and intensive exercises involving the affected muscle groups are needed (Lang et al. 2005; Yue et al. 2017).

The finger tapping task (FTT) is often used to assess explicit motor sequence learning (Buch et al. 2017). Basically, in the FTT, participants are asked to reproduce, as fast and accurately as possible, sequences of digit movements over repetitive sessions (Karni et al. 1995; Zimerman et al. 2012). This simple motor learning task is associated with both functional and structural changes in a wide distributed brain network, including: the primary motor (M1), the dorsal (PMd) and the ventral premotor (PMv) cortices, the supplementary motor area (SMA) and the posterior parietal cortex (PPC), as well as the cerebellum and the basal ganglia (e.g., Dahms et al. 2020). So far, very few efforts have been made to uncover whether and how explicit motor learning with a paretic limb is possible. In particular, a still open issue is whether a stroke affecting cortical motor areas, and/or their output efferent pathways, may impair basic motor learning mechanisms (Krakauer 2015). The present study addressed this issue in stroke patients with upper-limb hemiparesis, by assessing explicit online motor sequence learning in a sequential FTT executed with the paretic limb, and the strength of the retention of the new learned skill. We also took into account the role of clinical–demographic and neurological factors, as well as of lesion volume and location, on explicit motor learning performance.

## Materials and methods

### Participants

A series of 19 stroke patients, with contralateral upper-limb motor deficit, and no history or evidence of any other neurological or psychiatric disease, entered this study.

Patients gave their informed consent to the protocol, which was approved by the local Ethics Committees, and was conformed to the ethical standards of the 1964 Declaration of Helsinki.

The sample of patients included 5 females and 14 males with a mean age of 66 years (Standard Deviation, SD =  ± 12), a mean education of 11 years (± 5), and a mean time elapsed since stroke of 15.5 months (± 22). In 10 patients, the stroke affected the right cerebral hemisphere, nine patients had a left-sided brain damage. All patients had a normal or corrected-to-normal vision. According to a standard handedness self-report questionnaire (Oldfield 1971), one patient (with left lesion) was ambidextrous, 17 patients were right-handed, and one patient (with left lesion) was left-handed. Demographic and clinical details of the sample are summarized in Table 1. As assessed with standard neuropsychological batteries, no patients were diagnosed as having severe language comprehension deficits, unilateral spatial neglect, or upper-limb apraxia, which could compromise performance at the experimental task.

**Table 1:** Demographic and clinical data of patients

	ID	Gender	Age	Education (years)	Handedness	Aethiology	DUI (months)
	P1	M	60	13	R	I	5.5
Right BD	P2	M	47	8	R	I	4
	P3	M	56	18	R	I	1
	P4	M	68	13	R	I	3
	P5	M	80	5	R	I	1
	P6	F	79	5	R	I	2
	P7	F	75	5	R	I	2
	P8	M	76	13	R	I	2
	P9	M	60	17	R	H	3
	P10	M	55	8	R	H	36
Left BD	P11	M	72	18	R/L	H	53
	P12	F	33	18	R	I	11
	P13	M	67	13	R	I	3
	P14	F	81	8	L	I	60
	P15	F	73	13	R	I	63
	P16	M	69	19	R	H	5
	P17	M	56	6	R	I	12
	P18	M	69	5	R	I	2
	P19	M	72	13	R	I	1.5

*ID* Patients’ identification number, *Right BD* patients with right brain damage, *Left BD* patients with left brain damage. Gender: *M* male, *F* female. Handedness: *R* right-handed, *L* left-handed, *R/L* ambidextrous. Aetiology: *I* ischaemic stroke, *H*  haemorrhagic stroke. *DUI* duration of illness

Eighteen neurologically healthy subjects, with no history or evidence of neurologic or psychiatric disorders, were recruited to serve as the control group (CG). The sample included 13 females and 5 males with a mean age of 64 years (± 8), a mean education of 11 years (± 4.7). All participants had a normal or corrected-to-normal vision, with no history or evidence of psychiatric or neurological diseases. According to the handedness questionnaire (Oldfield 1971), all control participants were right-handed. The CG performed the motor task with the non-dominant left hand. Expert musicians were excluded. All participants gave their informed consent prior to their inclusion in the study, which was approved by the ethical committees of the IRCCS Istituto Auxologico Italiano and of Casa di Cura del Policlinico, Milan.

### Clinical assessment

As detailed in the following, the week before the experiment, patients underwent a comprehensive clinical assessment of their neurological status, particularly the upper-limb motor deficit; a reconstruction of the brain lesion (Rorden and Brett 2000) was also done (see “Lesion analysis” paragraph).

#### Neurological evaluation

The following two tests were administered:

(i) National Institute of Health Stroke Scale (NIHSS) (Brott et al. 1989). This is a 15-item scale evaluating the effects of stroke on consciousness, language, spatial attention, vision, motor strength (in this study only the upper limb was considered), ataxia, dysarthria, and sensory loss. Each item was scored with 3 or 5 grades, with 0 as normal, and 3/5 as severe impairment (Maximum total score, indicating most severe stroke = 34).
(ii) Assessment of unilateral visual half-field deficits (VFD) and extinction to double visual simultaneous stimulation, as assessed by the confrontation technique, and of somatosensory deficits (SSD) and extinction to double simultaneous stimulation (Bisiach and Faglioni 1974). For VFD and SSD the score ranged from 0 (unimpaired performance) to 3 (maximum deficit).

#### Motor evaluation

The following tests were administered (see Table 2):

**Table 2:** Motor data for patients

	ID	MI			MAL		FIM	BI	HG
		a	b	c	MAL-A	MAL-Q			(kg)
	P1	19	19	33	3	2	108	80	3
	P2	11	33	33	3	3	105	89	1.5
	P3	22	33	33	2	2	109	90	1.6
Right BD	P4	26	25	33	2	3	85	55	0.6
	P5	26	33	33	4	2	*n.a*	61	9.3
	P6	22	19	14	2	2	62	35	0.6
	P7	33	33	33	5	4	54	30	4
	P8	26	25	25	3	3	58	46	1.8
	P9	19	19	14	3	2	86	49	2.6
	P10	19	14	14	0	0	109	97	18.6
	P11	11	25	25	2	1	6	97	22
	P12	26	25	19	2	2	119	95	12.2
	P13	33	33	33	5	3	124	100	19
Left BD	P14	26	25	33	3	4	94	72	10
	P15	22	14	25	2	2	121	96	3.6
	P16	26	14	14	1	1	22	25	1.3
	P17	33	25	33	4	4	88	90	12.6
	P18	26	25	25	0	0	85	76	12.3
	P19	26	25	19	4	0	112	91	36.2

ID, right/left BD, see Table 1

*MI* Motricity Index (Range: 0–33): *a* pinch grip, *b* elbow flection, *c* shoulder abduction, *MAL* Motor Activity Log scale: *MAL-A* Amount of movements, quantitative subscale (score range = 0–5, 0 = not used; 5 = same as pre-stroke), *MAL-Q* Quality of movements, qualitative subscale (score range = 0–5, 0 = not used; 5 = normal). *FIM* Functional Independence Measure (range: 0–126). *BI* Barthel Index (range: 0–100). *HG* Hand Grip Strength Test (performance measured in Kg). *n.a.* not available

(i) *Motricity Index* (Demeurisse et al. 1980). This is a brief means of assessing motor impairment by examining pinch grip, elbow flection, and shoulder abduction. Each movement was given a score ranging between 0 = no movement to 33 = normal movement.
(ii) *Motor Activity Log scale, MAL* (Uswatte et al. 2006). In this semi-structured interview, patients were requested to record and evaluate the amount (subscale MAL-A) and quality (subscale MAL-Q) of daily life activities of the paretic arm, using a 6-point ordinal scale. Higher scores indicate better performance.
(iii) *Functional Independence Measure, FIM™* (Tesio et al. 2002). This is an assessment tool aimed at evaluating the functional status of patients in performing basic life activities safely and effectively. It comprised 18 items of tasks and assessed the patients’ need for assistance. Patients were asked to rate on a 7 points ordinal scale their need of assistance in performing a minimum set of skills related to self-care, sphincter control, transfers, locomotion, communication, and social cognition, from complete dependence to complete independence. Scores ranged from 18 (lowest) to 126 (highest) level of function.
(iv) *Modified Barthel Index* (Shah et al. 1989). This was an objective standardized tool for measuring functional status. Total scores ranged from 0 (complete dependence) to 100 (complete independence).
(v) *Hand grip strength test* (Mathiowetz et al. 1985). Patients were instructed to squeeze a dynamometer with the paretic hand as hard as possible, and hold it for 5 s. The average of three measurements (kg) was taken as a measure of strength.

### Lesion analysis

**Fig. 1:**
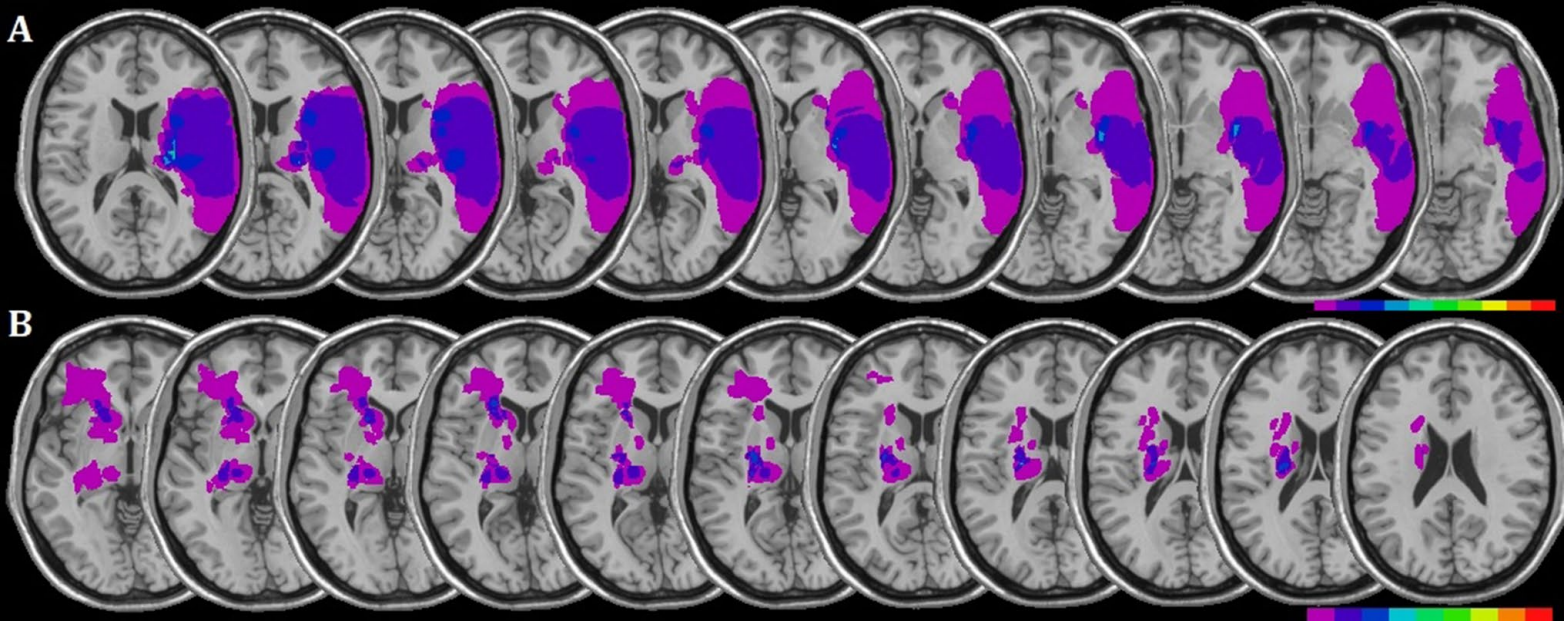
Lesions of patients. Overlay lesion plots for patients with a right-sided (**A**, *N* = 10) and left-sided (**B**, *N* = 9) brain damage. Each colour represents 20% increments, from red areas indicating maximum overlap, to pink areas indicating minimum overlap

MRI or CT scans were available for all patients. Regions of interest (ROIs) defined the location and the size of the lesion for each patient (Fig. 1). These were reconstructed by means of a template technique, by manually drawing the lesion on the standard template from the Montreal Neurological Institute (Rorden and Brett 2000), on each 2D slice of a 3D volume. Figure 1 shows the overlay lesion plot of all patients. Mean lesion volumes were 33.57 cc^3^ (± 62.41 cc^3^, range = 0.3–165.9 cc^3^) for patients with right brain damage, and 4.26 cc^3^ (± 4.76 cc^3^, range = 0.8–16.1 cc^3^) for patients with left brain damage.

### Explicit motor sequence learning task

The experimental task was an explicit sequential FFT (Karni et al. 1995; Zimerman et al. 2012).

**Fig. 2:**
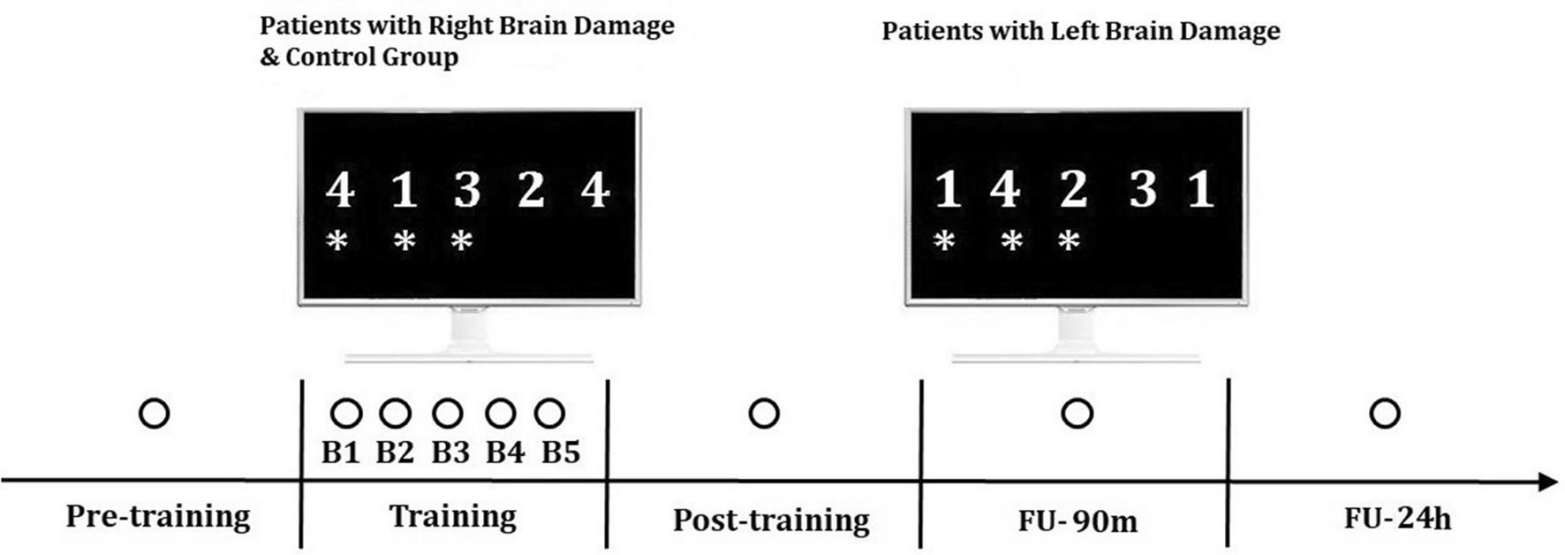
Schematic representation of the experimental design. Each circle represents a single block of 3 min each. The target sequence was presented before training (pre), repeated during the training phase (five blocks: B1, B2, B3, B4 and B5), and re-assessed immediately after (post), after 90 min (FU-90m), and after 24 h (FU-24h)

Participants had to perform a sequential digit pressing of a 5-element sequence on a 4-button keyboard with their hand, as quickly and accurately as possible. In particular, patients had to reproduce the sequence of numbers displayed over a computer screen with the corresponding fingers of their paretic hand, while healthy controls did the same with their left, non-dominant, hand. For patients with left brain damage, and thus with a motor deficit affecting their right hand, the following correspondence between fingers and numbers was used: index = 1, middle finger = 2, ring finger = 3, little finger = 4. Conversely, patients with right brain damage, and thus with a motor deficit affecting their left hand, the following correspondence was used: little = 1, ring = 2, middle = 3, and index finger = 4. The right-handed neurological healthy control participants performed the task with their left hand following the same digit/number correspondence applied for patients with right brain damage. Specifically, as depicted in Fig. 2, the sequence displayed over the computer screen was ‘4 1 3 2 4’ for controls and patients with right brain damage, and ‘1 4 2 3 1’ for patients with left brain damage. By applying the aforementioned correspondences between fingers and numbers, the two visually different sequences were comparable in terms of physical movements, namely all groups performed a series of index–little–middle–ring–index finger movements.

During the FTT, each stimulus was presented and controlled by PC software (E-prime 2.0 Psychology Software Tools), which also recorded the participants’ accuracy. An asterisk mark, appearing below the corresponding number, indicated task advancing after each button press, independently of the correctness of the typing. In case of errors, participants were asked not to correct, but to continue the task (e.g., Tecchio et al. 2010). No feedback regarding accuracy was provided. The task required to reproduce the entire 5-element sequence correctly; consequently, the performed sequence was incorrect if it contained even a single wrong press.

### Procedure

The main experimental session comprised 3 phases: pre-training, training, and post-training, for a total duration of about 30 min. There were also two follow-up (FU) sessions, each lasting 3 min. During each session, participants performed the same motor sequence (i.e., the target sequence). As shown in Fig. 2, during the pre-training phase, participants were presented with the sequence for a single block lasting 3 min. During the training phase, the participants’ task was to repeatedly perform the sequence (i.e., *online* learning) for five blocks of 3 min each, with 2 min breaks in between (e.g., Zimerman et al. 2012). Participants were required to reproduce again the sequence immediately after training (Post-training), after 90 min (FU-90m) and after 24 h (FU-24h), in all of these sessions for a single block of 3 min, to assess *retention*.

### Statistical analyses

Statistical analyses were performed using the IBM SPSS Statistics (Version 25). First, the normality of the data distribution was assessed by the Kolgorov–Smirnov test. Then, mixed measure analyses of variance (ANOVAs) were run to quantify motor sequence performance (*online* and *retention*). To this end, for each participant, the number of correct sequences reproduced within each block of the FTT was considered (Zimerman et al. 2012). *Online* motor sequence performance was analyzed via a mixed measure ANOVA, with the between-subjects factor *Group* (3 levels: RBD = Patients with right brain damage, LBD = Patients with left brain damage, CG = control group) and the within-subjects factor *Block* (5 levels: B1, B2, B3, B4, B5). *Retention* of the learned motor sequence was analyzed using the same ANOVA model, comprising the between-subjects factor *Group*, and the within-subjects factor *Time* (4 levels: Pre-training, Post-training, FU-90m, and FU-24h). In case of violation of the assumption of sphericity, Greenhouse–Geisser epsilon correction was applied. Significance was set at *alpha* = 0.05; main effects and interactions were further explored by means of Bonferroni correction.

We also explored the possible influence of baseline performance on learning effects by means of an analysis of covariance with the number of correct sequences before the training as a covariate. Accordingly, motor sequence performance was analyzed via a mixed measure ANOVA, with the between-subjects factor Group (3 levels: RBD = patients with right brain damage, LBD = patients with left brain damage, CG = control group), the within-subjects factor Blocks (B1, B2, B3, B4, B5) for online effects, and covariate pre-training performance. Similarly, a mixed measure ANOVA, with the between-subjects factor Group (3 levels: RBD = patients with right brain damage, LBD = patients with left brain damage, CG = control group), the within-subjects factor Time (post-training, FU-90m, FU-24h), and covariate pre-training performance was run to evaluate the effects on retention. Finally, further analyses were conducted considering the stroke patients, only. First, we computed a Motor Learning Index (MLI), using the following formula to calculate the percentage of the performance change (e.g., Bolognini et al. 2015):

$${\text{MLI}} = \frac{{{\text{B}}5 - {\text{B}}1}}{{{\text{B}}1}}{\text{\% }}{.}$$

Then, the MLI was used as dependent variable in a series of regression analyses, aimed at exploring the influence of the following variables (predictors) on *online* motor learning:

(i) clinical-demographic predictors: age, schooling and duration of illness;
(ii) neurological predictors: NIHSS score, presence of visual field (VFD) and somatosensory (SSD) deficit;
(iii) motor predictors: MI, MAL, FIM, BARTHEL and Hand Grip scores;
(iv) lesion predictors: total volume and number of lesioned voxels in each affected cortical area.

## Results

### Online learning

**Fig. 3:**
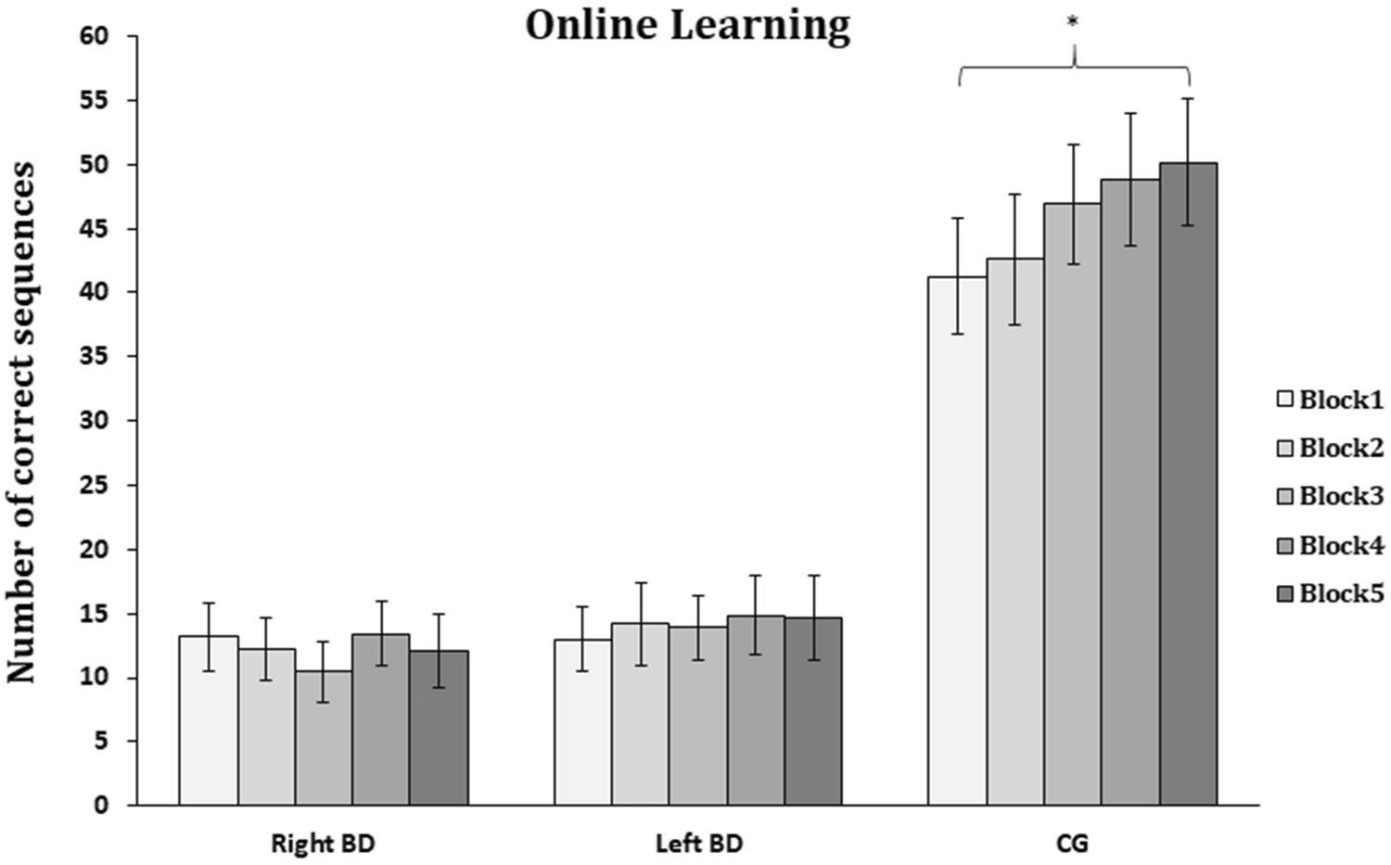
Online learning. Mean number of sequences correctly performed during each block of training for patients with right brain damage (Right BD) or left brain damage (Left BD), and in healthy controls (CG); *P < 0.05. Error bars = SE

Figure 3 illustrates the significant *Group X Blocks* interaction [*F*_(5.4,91.7)_ = 4.4, *P* < 0.01]. Specifically, only CG participants showed a significant amelioration of performance from the first (mean number of correct sequences, B1 = 41.3) to the last (B5 = 50.2; *P* < 0.01) block of practice; conversely no robust improvement was found in the two groups of patients, with right (B1 = 13.2 *vs*. B5 = 12.1; *P* = 1) and left (B1 = 13 *vs.* B5 = 14.7; *P* = 1) brain damage.

The ANOVA revealed also a significant main effect of *Group* [*F*_(2,34)_ = 20.7, *P* < 0.01], showing significant differences in the number of performed sequences during the 5-blocks task among CG (45.9) and patients with right (12.3, *P* < 0.01) and left (14.1, *P* < 0.01) brain damage, with no differences between the two groups of patients (*P* = 1). The main effect of *Block* was also significant [*F*_(2.7,91.7)_ = 4.7, *P* < 0.01], showing the overall improvement of motor performance during training.

Noteworthy the performances of both groups of patients in the first block of learning (B1) were significantly different from zero, as assessed by one-sample t tests (both *Ps* < 0.001), indicating that stroke participants could perform the task with their affected hand. Similarly, their performance in the last block of training (B5) remains significantly different from zero (*Ps* < 0.05 for both groups of patients).

### Influence of baseline performance on *online* effects

A significant effect of the main factors Blocks [*F*_(2.6,85.6)_ = 5.6, *P* < 0.01] and Group [*F*_(2,33)_ = 5.7, *P* < 0.01] was found, the last showing that controls (*M* = 33.7) performed overall a higher number of sequences, as compared to patients with right (23.3, *P* < 0.01), but not left (26.4, *P* = 0.09) brain damage. The two groups of patients did not differ between each other (*P* = 0.9). The interaction Blocks X Group approached significance [*F*_(5.2,85.6)_ = 2.2, *P* = 0.06], while the interaction Blocks X Baseline [*F*_(2.6,85.6)_ = 1.7, *P* = 0.18] did not reach the significance level, suggesting that baseline level of performance does not affected online motor learning.

### Retention

**Fig. 4:**
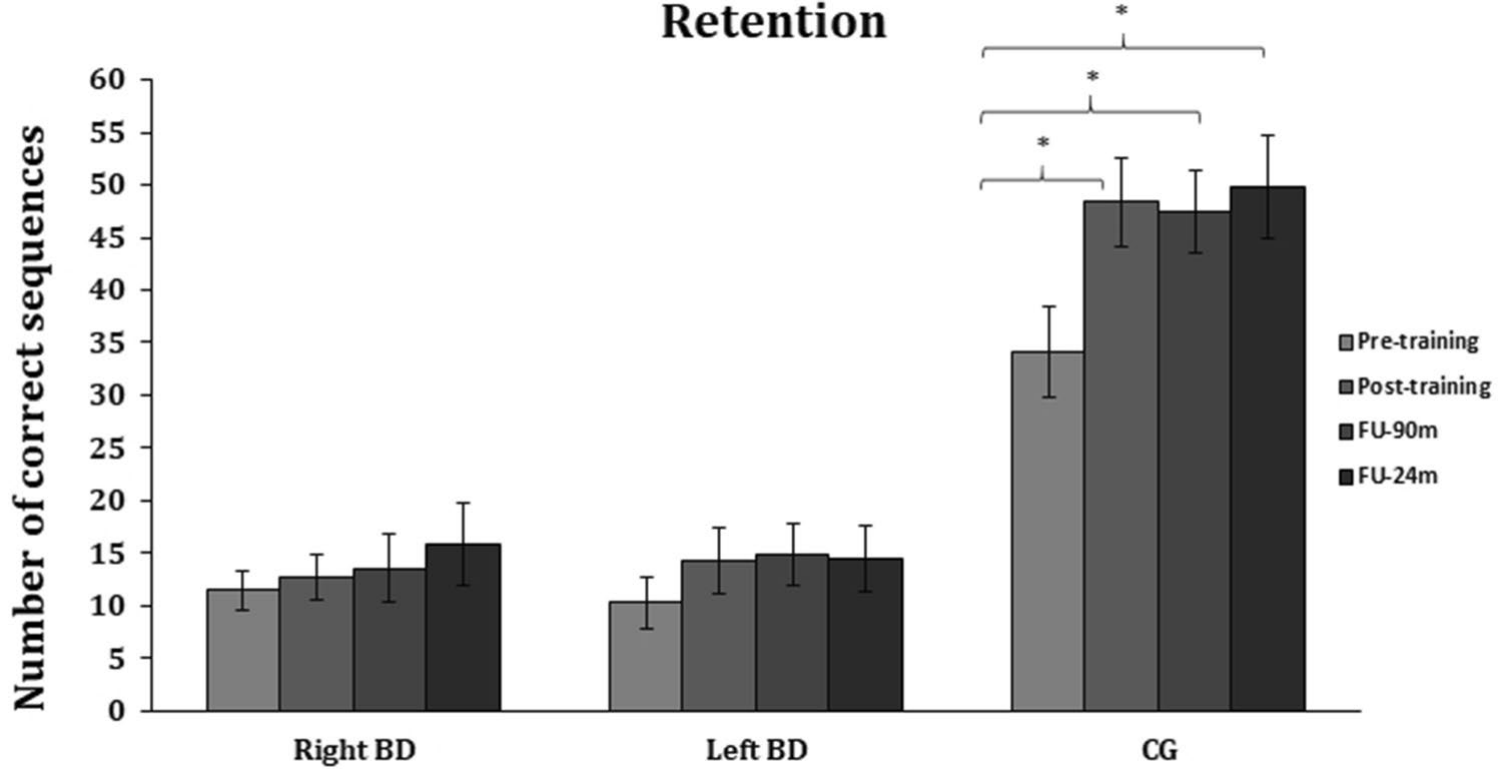
Retention. Mean number of sequences correctly performed during each block (3 min each) for patients with right brain damage (Right BD) or left brain damage (Left BD), and in healthy controls (CG); *P < 0.05. Error bars = SE

Figure 4 depicts the significant *Group* by* Time* interaction [*F*_(4.6,78.9)_ = 5.5, *P* < 0.01]**,** which showed an improved performance in the CG between the pre- (number of correct sequences = 34) and the post-training evaluations (post-training = 48.4, FU-90m = 47.5, FU-24h = 49.8, all *P*s < 0.01). Instead, both patients with right (pre-training = 11.5 *vs.* post-training = 12.7, FU-90m = 13.6, FU-24h = 15.9) and left (pre-training = 10.3, post-training = 14.3, FU-90m = 14.9, FU-24h = 14.4) brain damage did not show any improvement, as compared with pre-training performance (all *P*s < 0.6). Moreover, comparisons between the post-training and the follow-up evaluations showed a maintenance of the motor gains in CG (post-training *vs.* FU-90m and FU-24h, *P*s = 1). Performance did not change after the end of the training in both patients with right (post-training *vs.* FU-90m and FU-24h, Ps = 1) and left (post-training *vs.* FU-90m and FU-24h, *P*s = 1) brain damage.

The ANOVA showed also a significant main effect of *Group* [*F*_(2,34)_ = 24, *P* < 0.01], due to the higher performance of the CG (44.9) as compared to both patients with right (13.4, *P* < 0.001) and left (13.5, *P* < 0.01) brain damage, with no differences between the two groups of stroke participants (*P* = 1). The main effect of *Time* [*F*_(2.3,78.9)_ = 16, *P* < 0.01], showed an increased number of sequences correctly reproduced by each group of participants, immediately at the end of the 5-block training (post-training = 25.1), as well as at the 90m and 24h assessments (FU-90m = 25.3, FU-24h = 26.7, respectively; all *Ps* < 0.01), as compared to the baseline (pre-training) performance (18.6). No differences between the three post-training evaluations were found (*P*s = 1).

**Fig. 5:**
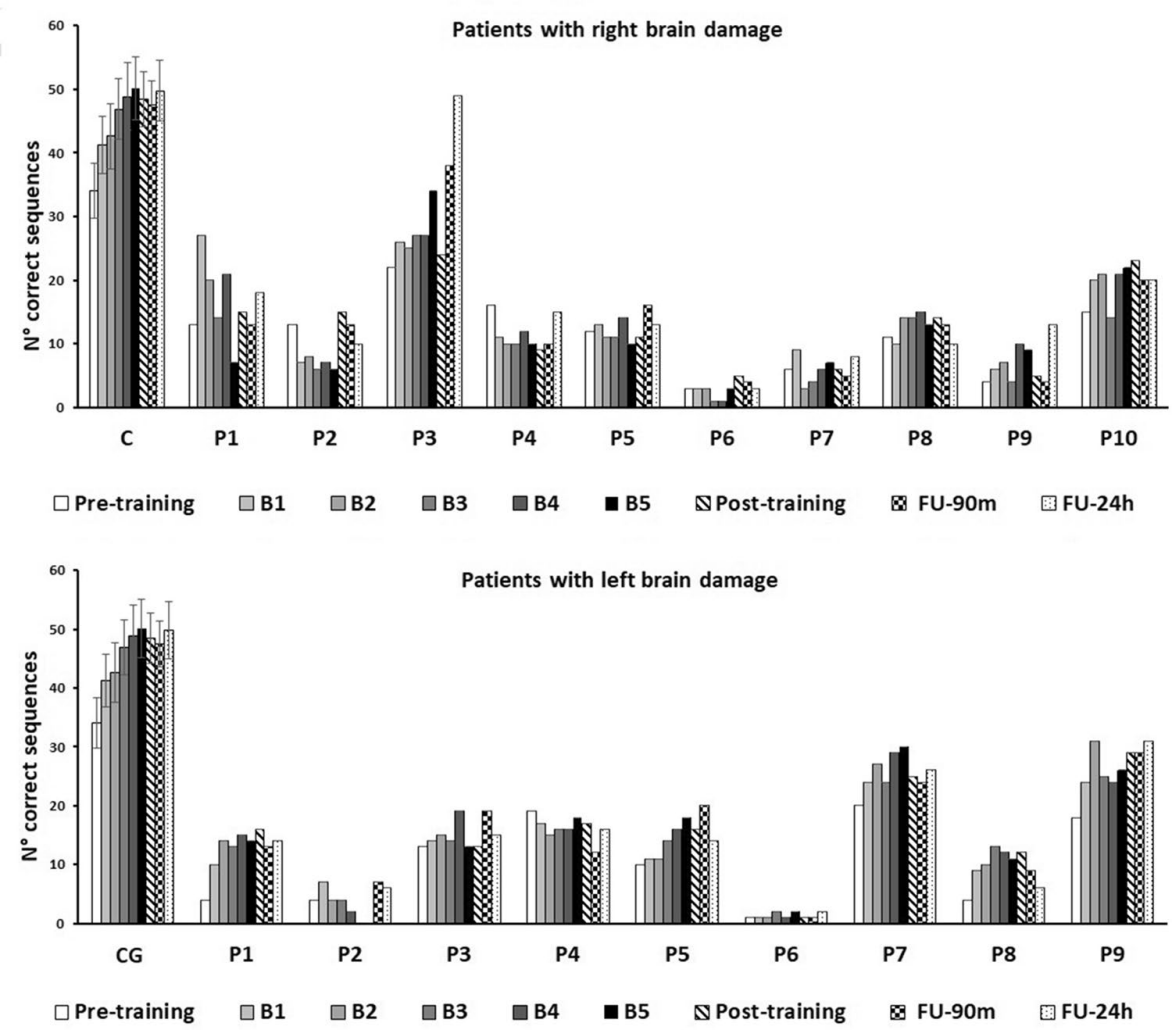
Individual data. Number of correct sequences performed during each time point for patients with right (top) and left (bottom) brain damage compared to the CG mean performance. Note that the patient P2 with a left brain damage (lower panel) was unable to perform any correct sequence in the last block of practice and in the post-training assessment

Individual data of each patient, for each time point, are shown in Fig. 5.

### Influence of baseline performance on *retention* effects

The analysis of covariance, with the baseline (pre-training) number of correct sequences as the covariate, showed a significant time × covariate interaction [*F*_(1.5,48)_ = 4.5, *P* = 0.03] suggesting that participants with a better baseline score exhibited greater stabilization of performance; conversely, participants with a poorer level of performance baseline improved less at the follow-up assessment. Time × Group [*F*_(2.9,48)_ = 1, *P* = 0.4], Time [*F*_(1.5,48)_ = 2.5, *P* = 0.1] were not significant. We also found a significant main effect of Group [*F*_(2,33)_ = 8.9, *P* < 0.01], showing that controls (*M* = 37.8) performed overall a higher number of sequences, as compared to patients with both right (23.7, *P* < 0.01) and left (25.2, *P* < 0.01) brain damage. The two groups of patients did not differ between each other (*P* = 1).

### Regression

Regression analyses with the MLI (see methods section) as dependent variable and clinical-demographic factors did not show any significant effect: age (*β* = 0.28, *t* = 1.1, *P* = 0.27) schooling (*β* = 0.27, *t* = 1 *P* = 0.29), duration of illness (*β* = 0.17, *t* = 0.72, *P* = 0.48).

Similarly, no relationship was found between the MLI and the following scores at the various neurological tests: NIHSS total (*β* = − 0.42, *t* = − 1.1, *P* = 0.29), VD score (*β* = − 0.14, *t* = − 0.38, *P* = 0.7), SD (*β* = − 0.08, *t* = − 0.24, *P* = 0.82), Motricity Index (*β* = − 0.26, *t* = − 0.61, *P* = 0.56), MAL_A (*β* = 0.21, *t* = − 0.34, *P* = 0.74), MAL_Q (*β* = − 0.44, *t* = − 0.56, *P* = 0.59), FIM (*β* = − 0.77, *t* = − 0.89, *P* = 0.4), Barthel Index (*β* = − 0.47, *t* = 0.48, *P* = 0.64), Hand Grip (*β* = − 0.4, *t* = − 0.65, *P* = 0.53). It should also be noted that there was no difference in all the above-mentioned tests between the two groups of patients (all *P*s > 0.05). The only exception was hand grip performance, with patients with right brain damage presenting a more severe strength deficit (*M* = 4.4 kg), as compared to patients with left BD (*M* = 14.3 kg) (*P* = 0.02).

**Fig. 6:**
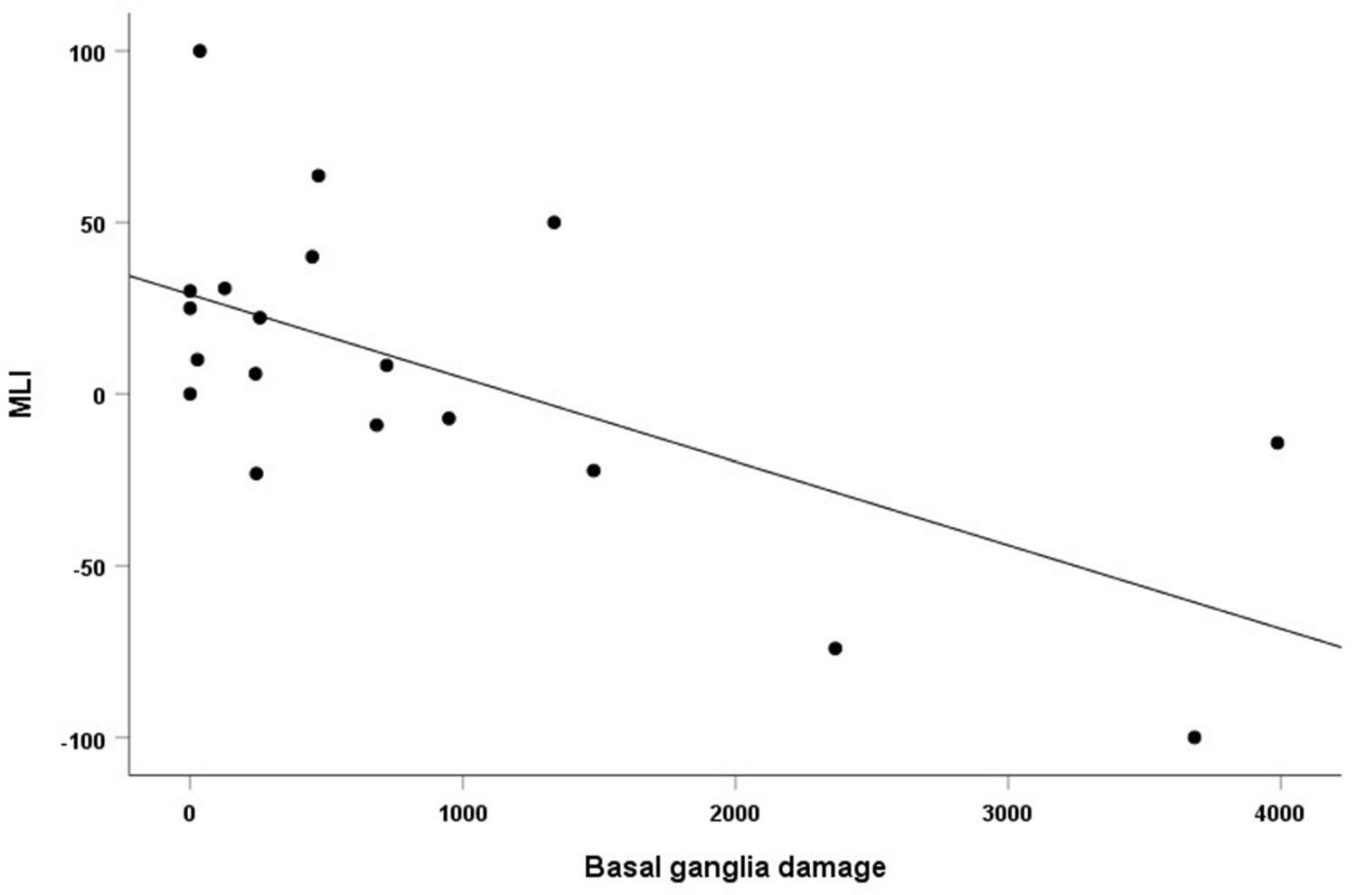
Scatterplot showing the relationship between MLI (Motor Learning Index) and basal ganglia lesion (number of lesioned voxels)

With respect to the lesion profile, although the overall lesion size (i.e., the total volume) did not predict the motor learning behavior (*β* = − 0.39, *t* = − 1.7, *P* = 0.1), some specific effects were found. The size (number of voxels) of the lesion affecting the basal ganglia was negatively associated with the MLI (*P* = 0.01), with the larger the damage of the basal ganglia, the lower the motor learning (i.e., the training improvement) [see Fig. 6]. No differences were found between the two groups of patients for both overall lesion size (*P* = 0.18), and amount of damage affecting the basal ganglia (*P* = 0.64).

## Discussion

The present study provides three main results. First, explicit motor sequence learning with the paretic hand is impaired in the majority of the stroke patients enrolled. Second, explicit motor sequence performance with the paretic hand in stroke patients is not influenced by demographical and clinical factors. Third, the amount of lesion affecting the basal ganglia predicts the amount of explicit motor sequence learning deficit in stroke patients.

Explicit motor sequence learning, as assessed through the FTT performed with the paretic hand, is featured by the absence of the typical practice-dependent progressive improvement in most of our stroke patients. A perusal of patients’ performances shows that some, but not all, do indeed show some motor gain, although to a minor extent as healthy controls. However, by taking into consideration the influence of baseline performance (i.e., pre-training level of performance) on online motor learning effects, it emerges that patients with right brain lesion performed worse than controls. Specifically, RBD patients executed a lower number of sequences during the training phase, as compared to the neurologically healthy participants, but they also present a more severe strength deficit than LBD patients. To note, RBD patients used their non-dominant (paretic) left hand to execute the task; this factor by itself probably explains their worse performance, rather than a specific motor learning impairment. As for retention, participants with a better baseline score exhibited greater stabilization of performance; conversely, participants with a poorer level of performance at baseline improved less at the follow-up assessments. Furthermore, in some cases, performance even deteriorate during the task, possibly because of motor fatigue. These results warrant future studies with bigger samples to further investigate patients’ features that influence motor learning capabilities. In our sample of stroke patients, the defective explicit motor sequence learning was not associated to demographic or clinical factors: age, time elapsed from stroke, stroke severity (NIHSS score), presence and severity of visual, somatosensory and motor deficits, do not account for the impairment in explicit motor learning performance.

The overall lower motor performance at the FTT of stroke patients, as compared to healthy controls, could be not surprising considering the evident upper-limb motor disorder which impacts on motor execution. Notwithstanding, it was not the motor deficit per se that prevented effective explicit motor learning. In fact, it is important to note that the capability of performing the task with the paretic hand was ascertained before delivering the experimental task: compatible with their upper-limb motor disorder, all patients were able to perform independent digit movement (i.e., move each finger from the index to the little finger in a separate manner) and had the necessary strength to deploy sufficient force to press the keys with all and each finger. Recently, it was shown that finger dexterity and explicit motor sequence learning are dissociable also in children affected by motor deficit due to unilateral cerebral palsy (Carneiro et al. 2020).

At first glance it could be speculated that post-stroke patients may need more training sessions to show learning effects. However, our findings are in line with evidence from a recent systematic review showing that (implicit) learning seems overall maintained for the unaffected hand, while it is absent for the paretic hand in stroke patients (Kal et al. 2016). Other evidence demonstrated an interference effect of explicit information in stroke patients. It has been proposed that the provision of explicit information after stroke may be less helpful than in neurologically healthy individuals for developing the motor plan, i.e., the discovery of a motor solution through practice and the reliance on the implicit system. Specifically, explicit information disrupted motor sequence learning after unilateral basal ganglia stroke and sensorimotor cortical strokes, regardless of the type of motor task, discrete (i.e., serial response task) or continuous (continuous tracking task) (Boyd and Winstein 2006). This could reflect an excessive working memory load in a system where functional and structural connectivity between the prefrontal cortex and motor regions is disrupted by the stroke (Boyd and Winstein 2004).

Our study represents a first attempt to address the often-neglected issue of stroke-related features in determining the efficacy of therapeutic interventions based on motor learning (Lefebvre and Liew 2017). A more in-depth comprehension of the factors underpinning post-stroke motor learning is indeed necessary to allow the identification of potential predictors to determine the effects of motor rehabilitation. In a recent study (Carneiro et al. 2020) explicit motor sequence learning was found to be impaired, as compared to the performance of age-matched neurologically healthy controls, in children with unilateral cerebral palsy with ipsilateral or contralateral corticospinal reorganization, but when the paretic hand was bilaterally controlled. This finding suggests that the motor learning performance of the paretic children was related to the type of reorganization of the corticospinal tract after early brain injury (Carneiro et al. 2020).

In this study, the lesion profile deserves particular attention, being in a neurological population a possible important source of heterogeneity, which may entail variability in the clinical outcome of post-stroke motor therapy. In healthy participants, variations in the functional and structural brain changes are related to the amount of motor skill learning. For instance, greater functional activation in the prefrontal, premotor, and parietal cortices, as well as in the basal ganglia and in the cerebellum, is associated with better learning (Tomassimi et al. 2011). In stroke patients, there is evidence that cortical activation during motor training features a recruitment of additional areas, such as the dorsolateral prefrontal cortex (DLPFC), as compared to the activation pattern of healthy individuals (Bosnell et al. 2011; Meehan et al. 2011). In addition, after stroke, practice-related changes are associated with a decreased activation in the undamaged hemisphere, along with an increased activity in the damaged hemisphere, compared to pre-training activation (Bosnell et al. 2011; Meehan et al. 2011). With respect to anatomical connectivity, in healthy participants, motor learning is positively associated with structural coherence in white matter tracts (Tomassimi et al. 2011). Similarly, after stroke, residual white matter connections within the affected hemisphere seem necessary for motor learning (Borich et al. 2014). Measures of white matter microstruttural status, such as fractional anisotropy (FA) indexed by diffusion tensor imaging (DTI), provide interesting evidence in this respect. Indeed, FA within the ipsilesional posterior limb of the internal capsule (PLIC) may represent a motor learning marker in chronic stroke, with patients presenting higher post-training FA in the ipsilesional PLIC showing greater implicit motor learning in a visuomotor pursuit task (Borich et al. 2014).

Previous evidence of the neural correlates of motor learning impairment is limited to implicit SRTT performed with the non-paretic hand. For instance, lesions of the premotor dorsal cortex are associated with an impairment of intentional retrieval of incidentally learned motor knowledge (Dovern et al. 2011). By focusing on participants with apraxia, the study by Dovern and coworkers (2011) showed that patients with left brain damage and limb apraxia are able to learn an implicit SRTT with their non-paretic hand in a similar manner to healthy controls and stroke patients without apraxia; however, they are impaired in intentionally retrieving the previously learned motor sequence. Such impairment is associated with lesions of the dorsal premotor frontal cortex (Dovern et al. 2011). In a later study, patients with damage to the left cerebral hemisphere were shown to be able to learn a complex sequence in a modified version of SRTT (in which both temporal and spatial components of the sequence were manipulated), while being impaired with purely temporal or purely spatial sequence learning (Dovern et al. 2016). Based on this pattern of results, the suggestion has been made that motor representations of a new learned task in patients with a left-sided hemispheric lesion are very fragile and prone to perturbation, with a performance decline over time (i.e., defective offline consolidation). However, it is important to note that this evidence pertains the non-affected, ipsilesional, hand (Dovern et al. 2016).

Other evidence showed that implicit motor sequence learning with the non-affected, ipsilesional, hand after a stroke in the sensorimotor cortical areas or basal ganglia does not benefit from the provision of explicit information. It follows that the basal ganglia and sensorimotor areas may play a pivotal role in influencing explicit task information’s role during sequence learning (Boyd and Winstein 2006). In the present study, using an explicit FTT performed with the paretic hand, we found a relationship between a damage affecting the basal ganglia and the abilities of explicit motor learning of patients with both right and left-sided hemispheric damage, namely: the greater the lesion affecting these nuclei, the greater the explicit motor sequence learning impairment. Basal ganglia (i.e., the striatum, formed by the caudate and putamen nuclei, the globus pallidus, the subthalamic nucleus and the substantia nigra) are a group of subcortical nuclei highly interconnected with frontal, prefrontal and parietal regions (Middleton and Strick 2000, 2002). Thanks to at least five discrete basal ganglia–thalamo–cortical circuits (Alexander et al. 1986), these structures may influence cognitive and motor functions (e.g., Helie et al. 2013). Studies in animals, healthy humans and post-stroke patients showed that the basal ganglia play a critical role in the planning and execution of a new motor skill, being also involved in higher order processes such as memory and motor learning (Groenewegen 2003; Packard and Knowlton 2002). Neurobiological models illustrate the role of the basal ganglia, in several motor learning sub-processes, from slow and fast learning to retention (Doyon and Benali 2005; Doyon et al. 2003). Online learning recruits the corpus striatum, over the involvement of the motor, prefrontal and parietal areas, the cerebellum and the hippocampus. After consolidation, the acquired new motor skill is represented within a wide network comprising cortico-cerebellar and cortico-striatal circuits (Doyon et al. 2009). The consolidation process is based mainly on a cortico-striatal loop circuit comprising parts of the basal ganglia (especially the putamen) and the premotor–motor cortex (Hikosaka et al. 1999). Moreover, these structures seem to play a key role in offline consolidation: basal ganglia activation (especially the putamen) increases after sleep following a motor sequence learning task (Debas et al. 2010).

Although the present study did not specifically address the involvement of cortico-striatal circuits and white matter networks in motor learning, our results reveal that lesions affecting basal ganglia inversely predict explicit motor learning abilities. This evidence suggests that damage to the basal ganglia, and, in turn, the potential breakdown of the involved cortico-subcortical circuits, may disrupt motor learning at several levels.

In line with such a model, patients with a stroke circumscribed to the basal ganglia exhibit an impaired motor learning (Boyd et al. 2009). In one study, patients with a subcortical damage due to stroke in the vascular territory of the middle cerebral artery underwent a SRTT with their non-paretic hand. By comparing the performance with repeated (i.e., learned and thus potentially planned in advance) and random sequences, it was found that patients with a stroke involving the basal ganglia do not group elements of the repeated sequence into functional motor units, at variance from healthy controls (Boyd et al. 2009). This finding is supported by further evidence in primates (Levesque et al. 2007; Tremblay et al. 2009) and humans (Boyd et al. 2009), suggesting the existence of a specific basal ganglia function in motor learning, namely the *chunking* process (Miller 1956). Lesions to basal ganglia may prevent patients to take advantage of this function, in turn impacting the learning a new task involving motor sequences (i.e., SRTT, see Dovern et al. 2016). Specifically, chunks in patients with basal ganglia lesions seem to be more rigid, more sensitive to disruptions and less adaptable than those in healthy controls (e.g., Dahms et al. 2020). Worth mentioning, motor learning is defective in patients with Parkinson’s disease, which affects basal ganglia circuits (Blandini et al. 2000). A recent meta-analysis indicates that patients with Parkinson’s disease are impaired in implicit motor sequence learning (i.e. SRTT, Hayes et al. 2015); however, these patients show learning deficits also in a modified version of the SRTT, in which the movement component is abolished (Vakil et al. 2000).

A main limitation of the present study is the small size of the sample. Studies involving larger samples of patients with post-stroke upper limb motor impairment are mandatory to confirm and extend the present evidence. Moreover, further investigations are warranted to explore in more detail the factors which may affect explicit motor learning after stroke*.* The present findings should be then regarded as preliminary, and should prompt a more in-depth investigation of other factors that may hinder or foster motor behavior in stroke patients, also for rehabilitation purposes. Future studies should also include other digit sequences, different from that presented during the practice, to assess generalization effects, clarifying whether improvements could be ascribed to a genuine skill-specific effect. A better understanding of residual motor learning capabilities of stroke survivors with upper-limb hemiparesis will optimize the translation of motor learning principles into rehabilitation procedures, thus guiding the development of more efficient therapeutic approaches aimed at post-stroke motor recovery.

In conclusion, the present study provides a novel evidence of explicit motor sequence learning deficits in patients with upper-limb hemiparesis, primarily associated to basal ganglia damage. Previous results were mainly based on the finding of an association between basal ganglia dysfunction and defective/abolished implicit motor learning, showing deficits in the consolidation phase after basal ganglia lesions (e.g., Dahms et al. 2020). Our study extends such evidence to the explicit learning of a motor sequence with the paretic hand.

## Data Availability

Data collected and analysed during the present study are available in the Open Science Framework (OSF) repository: https://osf.io/2su95/.
